# Genetic Background of Fetal Growth Restriction

**DOI:** 10.3390/ijms23010036

**Published:** 2021-12-21

**Authors:** Beata Anna Nowakowska, Katarzyna Pankiewicz, Urszula Nowacka, Magdalena Niemiec, Szymon Kozłowski, Tadeusz Issat

**Affiliations:** 1 Department of Medical Genetics, Institute of Mother and Child, Kasprzaka 17a, 01-211 Warsaw, Poland; magdalena.niemiec@imid.med.pl; 2 Department of Obstetrics and Gynecology, Institute of Mother and Child in Warsaw, Kasprzaka 17a, 01-211 Warsaw, Poland; ulasarzynska@gmail.com (U.N.); szymon.kozlowski@imid.med.pl (S.K.); tadeusz.issat@imid.med.pl (T.I.)

**Keywords:** fetal growth restriction, genetics, single nucleotide polymorphism, chromosomal microarray

## Abstract

Fetal growth restriction (FGR) is one of the most formidable challenges in present-day antenatal care. Pathological fetal growth is a well-known factor of not only in utero demise in the third trimester, but also postnatal morbidity and unfavorable developmental outcomes, including long-term sequalae such as metabolic diseases, diabetic mellitus or hypertension. In this review, the authors present the current state of knowledge about the genetic disturbances responsible for FGR diagnosis, divided into fetal, placental and maternal causes (including preeclampsia), as well as their impact on prenatal diagnostics, with particular attention on chromosomal microarray (CMA) and noninvasive prenatal testing technique (NIPT).

## 1. Introduction

Fetal growth restriction (FGR) is one of the most formidable challenges in antenatal care. While numerous formulae evaluating fetal growth have been established, the most common takes the head circumference (HC), abdominal circumference (AC) and femur length (FL) into account [1]. Although definitions of small for gestational age (SGA) and FGR are often used interchangeably, a clear distinction between those two must be made. A fetus is considered to be SGA when an estimated weight for a given gestational age falls below a preset threshold, usually the 10th centile [2]. A group of SGA fetuses does not necessarily contain unhealthy individuals; the majority of them are rather constitutionally small. On the other hand, a growth-restricted fetus is unable to reach its genetically programmed growth potential. Moreover, according to the newest criteria, an FGR fetus’ weight could even lie within normal ranges, and clear definitions have been established by Delphi consensus [2,3]. Nevertheless, both SGA and FGR should not be defined with rigid criteria, but rather considered as spectrum disorders [4].

Pathological fetal growth is a well-known factor of not only in-utero demise in the third trimester, but also postnatal morbidity and unfavorable developmental outcomes, including long-term sequalae such as metabolic disturbances, diabetic mellitus or hypertension [5,6,7]. In clinical considerations, two main types of FGR are often distinguished—early and late, i.e., diagnosed before or after 32 gestational weeks. Both types differ substantially, mostly in management, prevalence and pathophysiology. Although early FGR is associated with a higher risk of perinatal complication, late FGR is more common in clinical practice [2].

**Figure 1:**
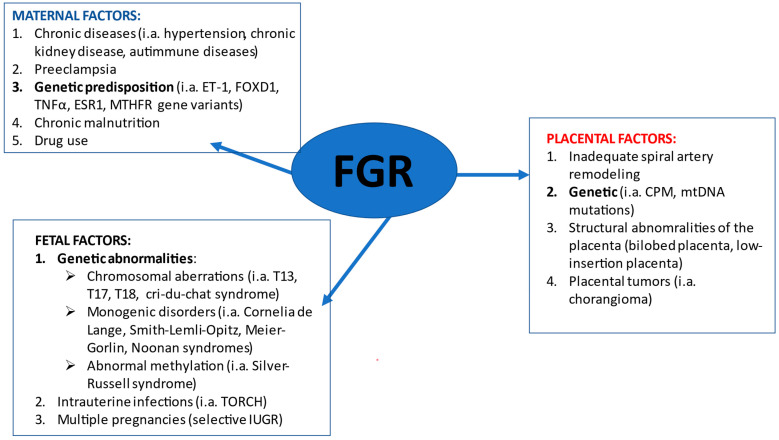
The most common factors involved in the pathophysiology of FGR.

Multiple factors playing roles in the pathophysiology of FGR have been reported. Nevertheless, impaired placentation remains as the main and most common culprit [2]. Recent development of diagnostic techniques allowed better analysis of genetic reasons for FGR, including chromosomal, submicroscopic and single gene disorders [8]. Although it is not a universal rule, genetic anomalies are more prevalent in severe cases and early gestational ages [8]. The genetic cause of FGR can be either fetal, maternal or impaired placental function [9]. The currently available genetic diagnostics, including the noninvasive prenatal testing technique (NIPT), as well as invasive sampling procedures (chorionic villus sampling CVS and amniocentesis) allow us to determine these abnormalities [10]. The most common factors involved in the pathophysiology of FGR are presented in Figure 1.

The primary objective of this narrative review was to explore to which extent genetic disturbances are responsible for FGR diagnosis. Since the genetic diagnosis determines the prognosis, it is of great importance to offer proper diagnostic testing. The secondary objective of this paper was to indicate the impact of possible genetic abnormalities on appropriate prenatal diagnostics and consequently on the prognosis and management strategies in women with FGR.

## 2. Fetal FGR Causes

The causes of fetal FGR vary [8] and include fetal chromosomal aberration, monogenic syndromes and abnormal methylation. Chromosomal anomalies were reported in up to 19% of fetuses with FGR, with aneuploidy being the most common aberrations, accounting for 5.8% [11,12]. Significant fetal growth restriction is often seen with trisomy 13 and trisomy 18, which are usually confirmed with multiple malformations by ultrasound. Similarly, FGR is the most common symptom of trisomy 17; however, the presence of this aberration is very rare [13]. The other genetic disorders with the same prenatal manifestation as FGR are Cri-du-chat syndrome (CdCS), resulting from a deletion of the short (*p*) arm of chromosome 5 [14,15,16], and Williams–Beuren syndrome, caused by a heterozygous deletion in the chromosome 7q11.23 region [17]. 

Recently, with the advancement of molecular genetic methods, the percentage of detected chromosomal aberrations has increased. The most recent non-invasive prenatal testing (NIPT), using cell-free fetal DNA in maternal blood, which is derived from the placental cytotrophoblast, was first designed for detecting fetal trisomies 21, 18 and 13. However, using a genome-wide approach, other rare chromosome anomalies can also be detected [10]. These aberrations were often “lost” during cell culture when performing a classical karyotype from trophoblast. Therefore, their frequency and clinical significance is now being investigated. In invasive prenatal diagnostics, using chromosomal microarray analysis (CMA), Tzadikevitch et al. revealed that the overall rate of chromosomal aberrations was significantly higher in all FGR cases when compared to the control group of normal uncomplicated pregnancies. The efficiency of CMA was also significantly higher in cases with major structural defects (29.4 vs. 3.4%, *p* = 0.001) compared to isolated FGR [18]. An and coauthors evaluated the incidence of chromosomal aberrations in a group consisting of 127 FGR cases without concomitant structural anomalies. In 9.4% (12/127) of cases with isolated FGR, chromosomal abnormalities were detected. Taking into account gestational age at diagnosis, the detection rate was 9.6% (5/52) in the early-onset group and 9.3% (7/75) in the late-onset group [19]. However, de Wit recommends performing CMA testing in cases of FGR between 18 and 24 weeks of gestation [20]. Peng et al. showed an inverse relationship between the gestational age at FGR diagnosis and the detection rate of chromosomal aberrations. It was 7% in the early-onset group and 1.8% in the late-onset group. An association between the chromosomal abnormality rate and FGR severity was also demonstrated: from 7.8% in fetuses with estimated fetal weight (EFW) below the 10th centile and 10% in those with EFW below the 5th centile, to 18% for fetuses with EFW below the 3rd centile [14]. A systematic review by Sagi-Dain et al. including 14 studies revealed a chromosomal abnormality rate of 6.4% (47/874) in isolated FGR cases [21]. 

When early-onset fetal growth restriction results from chromosomal abnormalities or is accompanied by fetal structural malformations, it may be associated with poor pregnancy outcomes. Nevertheless, the final pregnancy outcome depends also on the level of mosaicism. Chen et al. presented a case of low-level mosaicism for trisomy 16 in pregnancies complicated by early-onset FGR with a favorable outcome [22]. Gupta et al. found, however, an elevated risk of adverse pregnancy outcomes, even in patients with early-onset fetal growth restriction without fetal or genetic anomalies [23]. In a group of 122 patients, the incidence of intrauterine fetal death (IUFD) was 5.7%. High rates of other pregnancy complications, such as preterm birth (20%), low birth weight (59.3%) and gestational hypertension (14.1%), were also demonstrated. The prognosis worsens with early-onset and increased severity of growth restriction. Additionally, the presence of echogenic bowel and abnormal umbilical artery Doppler indices were associated with poor pregnancy outcomes of 17.4 versus 2.2%, OR 9.68, 95%CI 1.65–56.73 and 35.3 versus 0%, OR 4.46, 95%CI 2.65–7.50 respectively. [23]. 

FGR can also be a result of monogenic disorder. Variants in genes described in FGR cases are often associated with fetal growth, short stature or other skeletal anomalies (like skeletal dysplasia). Most of these syndromes will result in short stature, but many of them are additionally associated with intellectual disability [24]. Patients with FGR can be divided into two groups due to the clinical result of the mutation. The first group represents disorders where all fetal dimensions (such as head circumference, long bones) correspond to the same gestational age. This group includes, for example, the syndromes: Cornelia de Lange (most common mutation in the *NIPBL* gene), Smith–Lemli–Opitz (mutation in the *DHCR7* gene), Meier–Gorlin (mutation in the *ORC1*, *ORC4*, *ORC6*, *CDT1* or *CDC6* genes) or 3 M (most common mutation in the *CUL7* gene) [10,25,26,27]. The second group contains fetuses with extremely short long bones, but normal other dimensions and includes disorders such as Noonan Syndrome (most common mutation in the *PTP11* gene), achondroplasia or hypochondroplasia (mutation in the *FGFR3* gene) [10,28,29].

FGR is associated with an increased risk of developing metabolic diseases later in life [30]. Genetic variants in the *FTO* (fat mass and obesity-associated) and *PPARγ* (peroxisome proliferator-activated receptor-gamma) genes are known to be associated with the incidence of metabolic disease, body mass index and obesity. Barbieri et al. studied the association of selected *FTO* (rs1421085, rs55682395, rs17817449, rs8043757, rs9926289, and rs9939609) and *PPARγ* (rs10865710, rs17036263, rs35206526, rs1801282, rs28763894, rs41516544, rs62243567, rs3856806, and rs1805151) single-nucleotide polymorphisms (SNPs) with FGR in a Brazilian birth cohort. They revealed that *PPARγ* SNP was positively correlated and *FTO* SNP negatively correlated with FGR, and these effects were gender specific [31]. Karimi-Zarchi et al. suggested that the Apa1 polymorphism of insulin growth factor 2 (IGF-2) is associated with an increased risk of FGR [32]. Additionally, epigenetic changes, including i.a. DNA methylation, seems to be crucial in the origin of metabolic diseases. Krishna and coauthors compared the change in DNA methylation in umbilical cord blood samples of growth-restricted and appropriate for gestational age (AGA) babies to explore the differentially methylated candidate genes and their biological importance. Genomic DNA methylation varied significantly between FGR and AGA newborns (FGR: 3.12  ±  1.24; AGA: 4.40  ±  2.03; *p* value: <0.01), and a global shift toward hypomethylation was seen in FGR cases, targeted to regulatory regions and, specifically, promoters. As expected, pathway analysis identified dysregulation of the pathways responsible for the development of metabolic diseases [33]. One of the well-characterized diseases associated with FGR is Silver–Russel syndrome, which has multiple etiologies, but epigenetic changes in chromosome 11p15.5 and maternal UPD7 are the main causes of the syndrome [34,35].

## 3. Placental FGR Causes

Possible mechanism and diagnostic implications of placenta-limited genetic anomalies refers to the situation when the chromosomal anomaly can be confined to the placenta and cause growth restriction in a chromosomally normal fetus. Mosaicism is defined as the presence of two or more different chromosomal sets in the fetoplacental unit developed from a single zygote. It is caused by a viable somatic post-mitotic error occurring in an initially normal conceptus, or a meiotic error resulting in trisomy with subsequent post-zygotic trisomic rescue [36]. Placental mosaicism is observed in approximately 2% of analyzed chorionic villi samples and can engage different types of chromosomal abnormalities (structural or numerical) [37,38]. Especially in pregnancies with the isolated FGR, the possible mechanism of growth restriction is a presence of chromosomal abnormality limited to the placenta. In 9–16% of pregnancies with isolated FGR, chromosomal mosaicism confined to placental tissue (confined placental mosaicism, CPM) has been observed [39]. CPM can be divided into three types (I–III) and its type, as well as the particular chromosomal involvement and the origin of trisomy (mitotic or meiotic) in the aneuploid clone is related to specific pregnancy outcomes. When meiotic CPM with trisomy is detected in the placenta, there is a high risk of the fetus having uniparental disomy (UPD) for the chromosomal pair that is trisomic in the placenta [40,41]. Lazier et al. described maternal uniparental disomy of chromosome 6 [upd(6)mat] as a rare disorder with the main associated phenotype being FGR [42]. An additional special mention should be made for trisomy of chromosome 16, which is a cause of complication in more than 1% pregnancies [43]. However, CMP of chromosome 16 is associated with SGA in 43–58%, congenital malformation in 20–22%, preeclampsia 16–24% and prematurity in 37–37%. The normal outcome is observed in up to 36% of newborns [43,44,45]. Del Gobbo et al. presented a case of a liveborn male with FGR associated with confined placental mosaicism involving eight 2.4–3.9 Mb de novo duplications. These duplications were mosaic, detected not in the whole placenta, but only in its specific regions, and re-engaged both parental chromosomes, indicating a post-zygotic origin. Among the duplicated genes, there were some (including *KISS1* and *REN*) involved in trophoblast proliferation and angiogenesis and thus important for placental function and fetal growth [46]. CPM diagnosis not only explains the pathogenesis of FGR, but also ensures significant information for postnatal follow up. Thus, it is of importance to examine placentas from pregnancies with isolated FGR for the presence of genetic anomalies. This phenomenon is particularly visible, when the NIPT technique has been introduced on a large scale [47,48,49]. Van Opstal et al. performed NIPT in 2527 cases, and in 41 samples, chromosomal aneuploidies other than 13, 18 and 21 were detected. Among those 41, 22 were confined to the placenta. In 6 out of 22, FGR was observed, and in an additional four pregnancies SGA (small for gestational age (<p10)) was described [47]. Amniotic fluid sampling is regarded as the gold standard to differentiate true fetal mosaicism (TFM) from CPM, two conditions with a completely different prognosis [40,50,51,52]. Grati et al. reviewed 67,030 consecutive cytogenetic placental diagnoses, among which 1457 mosaic CVs were detected (2.17%). In 1100 of them amniocentesis was performed and TFM was identified in 13.5% of the cases (n = 148) [40]. In contrast to amniotic fluid, chorionic villi sampling is not relevant to CPM detection. Identifying mosaicism using this method is associated with FGR, pregnancy loss or IUFD only in some cases [37,41,53,54,55]. Clinical outcomes of CPM cover a wide range of results—from normal pregnancy to IUFD. The exact mechanism by which chromosomally abnormal cells in the placenta alter its function is unknown, but the effect seems to be related to specific chromosomes—especially chromosomes 2, 7–10, 13–18, 21, 22 containing genes important for fetal growth and placental function. These chromosomes have been demonstrated to be associated with adverse perinatal outcomes [39].

Alteration in gene expression in the placenta can cause abnormal fetal growth and development. More than 50 genes have been discovered to be necessary for fetal growth. One of the most important families of genes, essential for the proper functioning of the placenta, are Homeobox genes. These genes—*DLX3*, *DLX4*, *MSX2* and *GAX*, *ESX1L*, *TGIF-1* and *HLX1*—are expressed in the human placenta and play an important role in embryonic development. The downregulated expression of Homeobox genes *HLX1* and *ESX1L* (leading to proliferation, migration and invasion inhibition) and upregulated expression of homeobox genes *DLX3*, *DLX4* and *TGIF-1* (related to enhanced differentiation and apoptosis) has been found in idiopathic FGR cases [56,57,58]. *DLX3*, a transcription factor, interacts with the DNA-binding domain and the first transactivation domain of transcription factors glial cells missing 1 (GCM1), and this interaction inhibits GCM1-mediated placental growth factor expression [59]. Chelbi et al. measured SERPINA3 messenger RNA (mRNA) quantity in the placentas from normal and FGR pregnancies and showed that these levels were seven-fold higher in isolated FGR than in uncomplicated pregnancies [60]. Gascoin-Lachambre et al. divided the examined placentas in four groups: isolated preeclampsia (PE), PE associated with FGR, idiopathic FGR and vascular FGR. They demonstrated that the expression of *CUL1*, *CUL4A*, *CUL4B* and *CUL7* was significantly increased in pathological placentas in comparison to healthy controls, and its expression was position-dependent and necessary for early embryonic development. *CUL4B* and *CUL7* were particularly involved in placental-derived pathologies, including FGR [61]. Insulin-like growth factor 1 (IGF-1), IGF-2 and IGF-binding protein 3 (IGFBP-3) are also known to play an important role in intrauterine fetal growth. [62,63]. Börzsönyi B et al. performed a study aiming to evaluate gene expression patterns of IGF-1, IGF-2 and IGFBP-3 in human FGR placentas. They demonstrated significantly higher umbilical cord glucose and insulin levels in uncomplicated pregnancies when compared to FGR cases. There was an overexpression of IGF-2 and IGFBP-3 genes in FGR placentas (IGF-2: 1.67-fold expression, *p* = 0.04, IGFBP-3: 1.55-fold expression, *p* = 0.03). This overexpression of IGF-2 in the FGR placentas reflects its physiological role in optimizing energy distribution in a low-energy environment [64]. Additionally, Lee et al. presented significantly lower concentrations of glucose, insulin, IGF-1 and IGF-2 in the umbilical cord blood in FGR pregnancies. They also confirmed overexpression of IGF-2 and decreased IGF-1 expression in FGR placentas (*p* < 0.05) [65]. Giabacani et al. reported some new variants of IGF1R (coding type 1 insulin-like growth factor receptor) related i.a. to small for gestational age neonates [66].

There is also some evidence for the important role of placental mitochondrial DNA (mtDNA) alterations in the development of FGR. Using the whole mitochondrial genome sequencing, Naha et al. identified novel variants in both coding and non-coding regions of mtDNA in FGR placentas, performed Western blot analysis to determine the expression of mitochondrial Sirt3—a member of Sirtuin family proteins, responsible for maintaining mitochondrial homeostasis–and detected a significant increase in Sirt3 expression in FGR placentas. Additionally, a positive correlation between Sirt3 expression and birth weight in FGR cases has been demonstrated [67].

## 4. Maternal FGR Causes

Maternal variants, causing abnormal gene function, can also play a significant role in fetal FGR. An example can be endothelin 1 (ET-1)—the most potent vasoconstrictor. The pathogenesis of causing placental vasoconstriction is not known but enhanced contractile response of placental vessels to ET-1 has been proposed as a mechanism of FGR [68,69,70]. Arslan et al. in their trial enrolled 25 women with FGR pregnancy and 19 controls. The results of the study showed that the mean maternal ET-1 (FGR pregnancy 13.4 ± 6.2 vs. control 9.9 ± 2.9 pmol/L) and mean fetal ET-1 (FGR pregnancy 14.5 ± 4.2–11.7 ± 3.1 pmol/L) concentrations were significantly higher in FGR patients [71]. Quintero-Renderos et al. demonstrated Forkhead Box D1 (*FOXD1*) mutations’ central role in recurrent pregnancy loss (RPL), recurrent implantation failure (RIF), FGR and PE pathogenesis via complement C3 and placental growth factor (PlGF) regulation and described a functional link between *FOXD1* and implantation and placental diseases [72]. Another study suggested that the maternal -308G > A tumor necrosis factor α (TNF-α) gene variant may play a role in the development of FGR. It is known that in the placenta, TNF-α participates in the inflammatory response, induction of apoptosis, and cell proliferation, differentiation and migration [73]. Golovchenko et al. also identified haplotype TG (polymorphisms rs2234693-rs9340799) of the estrogen receptor α (ESR1) gene as a risk factor for developing FGR [74]. ESR1 is a ligand-activated transcription factor indispensable for appropriate hormone binding. During pregnancy, estrogen stimulates i.a. the activation of IGF-1 receptor, promoting fetal growth and response to different signals, such as hypoxia, hormones and nutrients [63,75]. Although in the last few years a lot of studies have been published concerning the potential association of methylenetetrahydrofolate reductase (MTHFR) 677C > T polymorphism with the risk of FGR and placental abruption, they didn’t bring enough evidence to support introducing MTHFR genetic testing into routine clinical practice [76,77,78]. The meta-analysis of case-control studies published in 2020 provided, however, moderate to strong evidence for the association between MTHFR 677C > T polymorphism and increased risk of developing FGR and placental abruption, especially in Caucasians and Africans [79].

The most common genetic causes of FGR are summarized in Table 1.

## 5. Fetal Growth Restriction and Preeclampsia as a Maternal Factor

PE is defined according to The International Society for the Study of Hypertension in Pregnancy (ISSHP) as the presence of a new-onset hypertension after 20 weeks of gestation accompanied by proteinuria or evidence of maternal acute kidney injury, liver dysfunction, neurological features, hemolysis or thrombocytopenia, or FGR [80]. In particular, early-onset preeclampsia is very often accompanied by FGR. In a study performed by Hung et al., FGR was diagnosed in 50.6% of women with early-onset PE and in 25.5% patients with late-onset disease [81]. Such a high incidence of FGR during PE is related to the very similar pathomechanism of these two pregnancy complications, based on the inadequate trophoblast invasion into maternal spiral arteries and maternal endothelial dysfunction [82,83]. However, it is still under consideration why among women with the same risk factors, some of them develop only FGR and some of them develop PE with or without FGR. 

The role of genetic factors in PE is undeniable because of the family history of the disease. Despite this, large genome-wide association studies (GWAS) performed in recent years have not brought expected results [84]. In the largest GWAS study in PE consisting of the offspring of 4380 PE patients and 310,238 controls, the only SNP in the fetal genome associated with the PE risk was the SNP in *FLT1* gene, coding soluble fms-like tyrosine kinase 1 (sFlt-1)—a crucial antiangiogenic factor involved in PE pathophysiology [85]. The largest PE GWAS study in the maternal genome was the HAPO study (hyperglycaemia and adverse pregnancy outcome), but in its results no individual SNPs were significantly associated with PE, despite over one million SNPs being assayed [86]. However, there are some case-control observational studies indicating the possible relationship of different polymorphisms with PE and FGR. The relationship of Storkhead Box 1 (*STOX1*) gene (involved in the differentiation of proliferative trophoblast into invasive type) with the family history of PE was confirmed in the Danish population [87]. In a meta-analysis performed in 2018, the only detected correlation with PE risk was 4G/5G polymorphism of plasminogen activator inhibitor 1 (PAI-1) [88,89]. Gannoun et al. investigated genetic polymorphisms in the promoter region of matrix metalloproteinases 9 and 2 (MMP-9 and -2) in a Tunisian Arab population and revealed that the MMP-9 rs3918242 (-562 C > T) variant is related to increased MMP-9 production and is a risk factor for PE [90]. In a recently published meta-analysis, Liu et al. identified transforming growth factor-beta 1(TGF-β1) rs800469 variant as a possible risk factor for PE in Asian population [91]. TGF-β1 is a cytokine produced during pregnancy by trophoblast cells, involved in the regulation of trophoblast proliferation, differentiation and invasion. Plasma TGF-β1 levels, as well as its expression in the placenta, are significantly higher in PE patients than in healthy controls [92]. In another meta-analysis, concerning genetic variations in other cytokines: intrelukin-1 and -6 (IL-1 and IL-6), it was demonstrated that two IL-β1 polymorphisms (rs1143634 and rs16944) are related to the risk of developing PE during pregnancy [93]. Additionally, Mazlum et al. showed that the IL-32 rs4786370 variant was related to elevated serum levels of IL-32 in women with PE and thus can be regarded as a risk factor for PE [94]. PE is often associated with proteinuria and renal injury, most of all affecting the number and function of podocytes, the cell population crucial in appropriate glomerular filtration [95]. Khaliq et al. investigated polymorphisms in two genes important for renal function: actin binding protein 4 (ACTN4) and nephrin (NPHS1). It is known that individuals with a mutation in the ACTN4 gene have clinical signs of nephrotic syndrome. Similarly, patients with NPHS1 mutation suffer from renal injury. This study revealed that the C allele of rs437168 NPHS1 is significantly associated with early-onset PE, whereas there was no association of ACTN4 polymorphisms with the risk of PE [96]. Ma et al. indicated the TC 96121524 variant of endoplasmic reticulum aminopeptodase-1 (ERAP-1) gene as potential risk factor for developing PE, where endoplasmic reticulum stress is one of the well-documented processes underlying PE pathophysiology [97,98]. The other very important factor in the pathophysiology of PE is the role of nitric oxide (NO)—the major vasodilator involved in the regulation of arterial blood pressure. Studies on animal models have shown the crucial role of the NO/NO synthase (NOS) system for normal spiral artery remodeling [99,100]. During uncomplicated pregnancy, vascular endothelial growth factor (VEGF) increases the expression of endothelial NOS (eNOS) and activates it. In PE and FGR pregnancies, excessive production of sFlt-1 leads to the binding of VEGF and thus to the lack of appropriate eNOS activity [101]. Jakovljevic et al. revealed that -786 T/C and VNTR 4b/a eNOS gene polymorphisms were associated with PE risk [102]. A recently published meta-analysis confirmed that the c.894 T (p.298Asp) and g.-786C alleles of eNOS gene may influence the NO bioavailability and were significantly associated with PE [103].

The impact of different polymorphisms and genotypes on the risk of developing FGR and PE is summarized in Table 2.

## 6. Summary

The results showed that an invasive prenatal procedure is strongly recommended when FGR is diagnosed. CMA detects all the chromosomal aberration detected by karyotyping and has a 5.5% additional detection rate of a genetic cause of FGR, which could impact the clinical decision. CMA as the first-line test is thus effective and feasible as a joined prenatal testing for suspected FGR cases. Additionally, a recent advantage in molecular methods enables investigation of single nucleoid variants in many genes simultaneously. Few gene panels have been designed for FGR investigation, which include from 25 up to 44 genes involved in fetal growth restriction. Additionally, the introduction of exome sequencing gives the possibility of investigating all coding regions in one test; however, the diagnostic rate in FGR cases remains unknown. Recently, a promising method was successfully developed for assessing the risk of FGR at an early gestational age based on the maternal plasma cfDNA nucleosome profiling. Through the whole-genome sequencing data analysis of FGR cases and controls, genes with significantly differential DNA coverage at promoter regions were identified and the non-invasive ‘FGR classifier 1’, based on 14 genes with differential promoter coverage, was developed [104].

Genetic testing in FGR cases is particularly valuable not only in cases with concomitant structural malformations, but also in isolated FGR. The detection of genetic abnormalities in such cases significantly affects the prognosis and management strategy, as well as the postnatal care of the growth-restricted newborn.

## Figures and Tables

**Table 1:** Most common causes of FGR.

Origin	Type of Genetic Abnormality	Examples	Referenes
Fetal	Chromosomal aberrations	Trisomy 13, 17 and 18	[11,12,13]
		Cri-du-chat syndrome (5p15.2 or 5p15.3 deletion)	[14,15,16]
		Williams–Beuren syndrome (7q11.23 deletion)	[17]
	Monogenic syndromes	Cornelia de Lange syndrome (*NIPBL* mutation)	[10,25,26,27]
		Smith–Lemli–Opitz syndrome (*DHCR7* mutation)	
		Meier–Gorlin syndrome (*ORC1*, *ORC4*, *ORC6*, *CDT1* or *CDC6* mutation)	
		3 M syndrome (*CUL7* mutation)	
		Noonan syndrome (*PTP11* mutation)	
		Achondroplasia or Hypochondroplasia syndrome (*FGFR3* mutation)	[10,28,29]
	Abnormal methylation	Silver–Russel syndrome (11p15.5 epigenetic changes)	[34,35]
Placental	Confined placental mosaicism	Most common chromosomes: 2, 6, 7–10, 13–18, 21, 22	[36,37,38,39,40,41,42,43,44,45,46,47,48,49,50,51,52,53,54,55]
	Alterations in gene expression	Upregulation	
		*DLX3* and 4	[56,57,58]
		*TGIF-1*	
		*HLX1*	
		*CUL1*	[61]
		*CUL4B*, *4a*	
		*CUL7*	
		IGF-2 and IGFBP-3	[62,63,64,65]
		Downregulation	
		*ESX1L*	[56,57,58]
		*HLX1*	
		IGF-1	[65]
	Single nucleotide variants	*IGF1R*	[66]
	Mitochondrial increased expression	Sirtuin-3	[67]
Maternal	Gene mutations causing its abnormal function	ET-1 *FOXD1* TNFα	[68,69,70,71] [72] [73]
	Single nucleotide polymorphisms	rs2234693-rs9340799 ESR1 variants MTHFR 677C > T variant	[74,75] [76,77,78,79]
	Genetic causes of preeclampsia		

IGF-1—insulin growth factor 1; IGF-2—insulin growth factor 2; IGFBP-3—IGF-binding protein 3; IGF1R—insulin-like growth factor receptor type 1; ET-1—endothelin 1; TNFα—tumor necrosis factor α; ESR1—estrogen receptor α; MTHFR—methylenetetrahydrofolate reductase.

**Table 2:** The impact of different polymorphisms and genotypes on risk of developing FGR and PE.

Gene	Variant/Genotype	Effect Size	References
FGR			
*TNF* *α*	-308GA	OR 1.65 (95% CI 0.93–2.93) *p* = 0.06 OR 2.8 *p* = 0.01 with uterine arteries flow abnormatlities	[72]
*ESR1*	rs2234693/rs9340799 TG genotype	OR 1.94 *p* = 0.001	[73]
*MTHFR*	677C > T TT + TC vs. CC overall Caucasian ethnicity African ethnicity	OR 0.14 (95% CI 0.049–0.045) *p* < 0.001 OR 0.15 (95% CI 0.04–0.561) *p* = 0.005 OR 0.162 (95% CI 0.06–0.436) *p* < 0.001	[78]
PE			
*FLT1*	rs4769613 C allele (fetal genome)	OR 1.22 (95% CI 1.14–1.31)	[84]
*PAI-1*	4G/5G	OR 1.36 (95% CI 1.13–1.64)	[88]
*MMP-9*	rs3918242 (-562C > T)	OR 1.62 (95% CI 1.03–2.56)	[89]
*TGF* *β1*	rs1800469	OR 1.17 (95% CI 1.02–1.35) OR 1.35 (95% CI 1.06–1.72) OR 1.48 (95% CI 1.07–2.05) Allele, recessive and homozygous model respectively	[90]
*IL-1* *β*	rs1143634 T allele	OR 1.28 (95% CI 1.04–1.58)	[92]
*NPHS1*	rs437168 C allele C vs. T CC vs. CT/TT	OR 1.287 (95% CI 1.021–1.622) OR 1.398 (95% CI 1.005–1.945)	[95]
*ERAP-1*	96121524 TC genotype	OR 2.002 (95% CI 0.687–5.831) *p* = 0.02	[96]
*eNOS*	-86T/C (CC variant)	*p* = 0.006 PE *p* = 0.01 9 early onset PE *p* = 0.012 late onset PE	[101]
	VNTR 4a4a homozygote	OR 7.68 (95% CI 0.89–65.98) *p* = 0.04	[101]
*IL-32*	rs4786370 T allele CT/GT TT/TT TT/GT	OR 2.75 (95% CI 1.34–5.642) OR 3.266 (95% CI 1.279–8.339) OR 3.438 (95% CI 1.354–8.73)	[93]

TNFα—tumor necrosis factor α; ESR1—estrogen receptor α; MTHFR—methylenotetrahydrofolate reductase; PAI-1—plasminogen activator inhibitor 1; MMP-9—matrix metalloproteinase 9; TGFβ1—transforming growth factor β1; NPHS1—nephrin; ERAP-1—endoplasmic reticulum aminopeptidase-1; eNOS—endothelial nitric oxide synthase; IL-32—interleukin 32.

## Data Availability

No new data were created or analyzed in this study. Data sharing is not applicable to this article.
